# Prospektive Vergleichsanalyse von CI-Patienten mit einseitiger Taubheit und asymmetrischem Hörverlust hinsichtlich der gesundheitsbezogenen Lebensqualität, Tinnitusbelastung und psychischen Komorbiditäten

**DOI:** 10.1007/s00106-023-01318-6

**Published:** 2023-07-12

**Authors:** Mohamed Bassiouni, Sophia Marie Häußler, Manuel Christoph Ketterer, Agnieszka J. Szczepek, Jana Vater, Lynn Hildebrandt, Moritz Gröschel, Heidi Olze

**Affiliations:** 1 Klinik für Hals-Nasen-Ohrenheilkunde, Charité - Universitätsmedizin Berlin, Augustenburger Platz 1, 13353 Berlin, Deutschland; 2 Klinik für Hals-Nasen-Ohrenheilkunde, Universitätsklinikum Hamburg Eppendorf, Martinistr. 52, 20246 Hamburg, Deutschland; 3 Klinik für Hals‑, Nasen- und Ohrenheilkunde, Universitätsklinikum Freiburg, Killianstr. 5, 79106 Freiburg, Deutschland

**Keywords:** Hörstörungen, Sprachaudiometrie, Hörhilfen, Hörrehabilitation, Cochlea Implantat, Hearing disorders, Speech audiometry, Hearing aids, Hearing rehabilitation, Cochlear implant

## Abstract

**Hintergrund:**

Patient:innen mit einseitiger Taubheit („single-sided deafness“, SSD) und asymmetrischem Hörverlust („asymmetric hearing loss“, AHL) werden zunehmend mit Cochleaimplantaten (CI) versorgt, da eine Verbesserung der auditiven Fähigkeiten und der Lebensqualität nachgewiesen wurde. Bisher gibt es nur wenige Veröffentlichungen, in denen die beiden Gruppen vergleichend untersucht werden. Ziel der vorliegenden Studie war es zu prüfen, worin sich diese beiden Gruppen, insbesondere präoperativ, unterscheiden.

**Methodik:**

Es handelt sich um eine statistische Sekundäranalyse bereits veröffentlichter Rohdaten von 66 CI-Patient:innen (21 SSD/45 AHL), die prospektiv in die Studie eingeschlossen wurden. Neben dem Sprachverstehen wurden Tinnitusbelastung (Tinnitusfragebogen), krankheitsspezifische Lebensqualität (Nijmegen Cochlear Implant Questionnaire, NCIQ), Stressbelastung (Perceived Stress Questionnaire, PSQ) und psychische Komorbiditäten (Allgemeine Depressionsskala, ADS‑L, und Generalized-Anxiety-Disorder-Fragebogen, GAD-7) bei SSD- und AHL-Patient:innen prä- und postoperativ verglichen.

**Ergebnisse:**

Präoperativ zeigte die SSD-Gruppe im NCIQ in den Subdomänen elementare und erweiterte Schallwahrnehmung signifikant höhere Werte als die AHL-Gruppe. Die Stressbelastung (PSQ) und die Angstsymptomatik (GAD-7) waren bei SSD-Patienten signifikant höher als bei AHL-Patient:innen. Diese Unterschiede waren 6 Monate postoperativ stark verringert und teils sogar nicht mehr signifikant.

**Schlussfolgerung:**

Präoperativ unterscheiden sich SSD- und AHL-Patient:innen signifikant im Hinblick auf die subjektive Höreinschätzung und psychosoziale Parameter. Bei SSD-Patient:innen können psychische Belastungsfaktoren einen stärkeren Einfluss auf die gesundheitsbezogene Lebensqualität haben als bei AHL-Patient:innen. Diese Aspekte sollten in der präoperativen Beratung sowie in der postoperativen CI-Rehabilitation berücksichtigt werden.

## Abstract (en)

**Background:**

Patients with single-sided deafness (SSD) and asymmetric hearing loss (AHL) are increasingly being treated with cochlear implants (CI) due to the demonstrated improvements in auditory abilities and quality of life. To date, there are few published studies in which these two groups are comparatively studied. The aim of the current study was to examine which factors differ between those two patient groups, especially preoperatively.

**Methods:**

A secondary analysis of the previously published raw data of 66 prospectively recruited CI patients (21 SSD/45 AHL) was performed. In addition to the hearing outcome, tinnitus distress (tinnitus questionnaire), health-related quality of life (Nijmegen Cochlear Implant Questionnaire, NCIQ), stress (Perceived Stress Questionnaire, PSQ), and psychological comorbidities (General Depression Scale, ADS‑L and Generalized Anxiety Disorder scale, GAD-7) were assessed in SSD and AHL patients pre- and postoperatively.

**Results:**

Preoperatively, SSD patients showed significantly higher scores in the NCIQ subdomains “elementary” and “advanced sound perception” than the AHL group. Stress (PSQ) and anxiety symptoms (GAD-7) were significantly higher preoperatively in SSD patients than in AHL patients. After CI, these differences were strongly reduced, with minimal differences being detectable between the groups in the investigated domains postoperatively.

**Conclusion:**

SSD and AHL patients differ significantly preoperatively in terms of their subjective hearing assessment and psychosocial parameters. In SSD patients, psychological stress factors may have a stronger impact on the quality of life than in AHL patients. These aspects should be taken into account in the preoperative counseling and postoperative rehabilitation.

## Hintergrund

Bei hochgradiger, an Taubheit grenzender Schwerhörigkeit ermöglicht die Versorgung mit einem Cochleaimplantat (CI) ein besseres Sprachverstehen als mit konventionellen Hörgeräten [1]. Für die CI-Versorgung von postlingual ertaubten Erwachsenen schlägt die S2k-Leitlinie als Schwelle ein monaurales Sprachverständnis im Freiburger Einsilbertest von ≤ 60 % bei 65 dB trotz optimaler Hörgeräteversorgung vor [1]. Der Großteil der bisherigen Studien bezieht sich hierbei auf beidseitigen Hörverlust („double-sided deafness“; DSD) [2, 3]. Allerdings wird die Versorgung von Patient:innen mit einseitiger Taubheit („single-sided deafness“; SSD) und asymmetrischem Hörverlust („asymmetric hearing loss“; AHL) ebenfalls empfohlen, da nicht nur eine Verbesserung des Sprachverstehens, sondern auch der Lebensqualität und der Tinnitusbelastung in einer Vielzahl von prospektiven und retrospektiven Studien nachgewiesen wurde [4–13]. Bei AHL wird das kontralaterale Ohr mit einem Hörgerät unterstützt, sodass eine bimodale Versorgung ein binaurales Hören ermöglicht, wie es sich auch bei SSD und CI ergibt. Binaurales Hören bezieht sich auf das Phänomen, bei dem die Patient:innen Schallinformationen aus beiden Ohren wahrnehmen.

**Figure 1:**
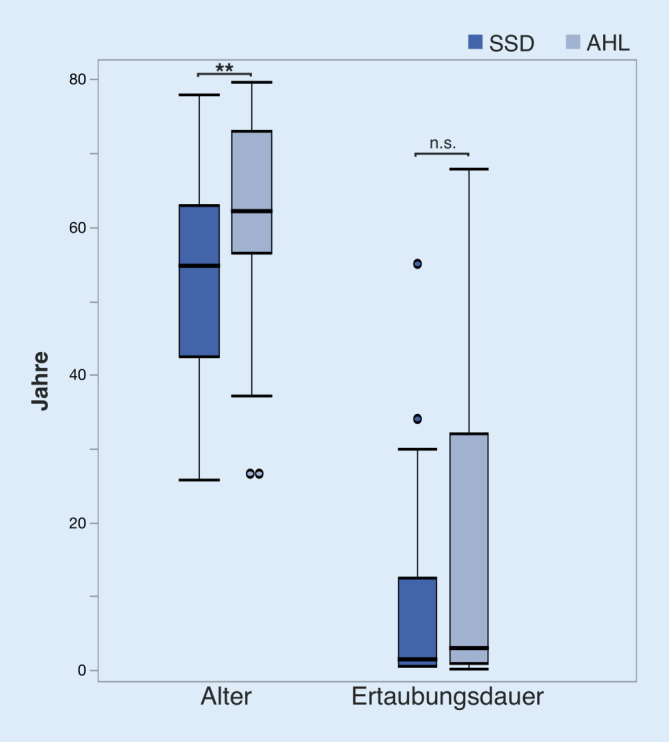

Schwerhörigkeit hat allgemein einen enorm starken negativen Einfluss auf die subjektive Lebensqualität [14]. In vorhergehenden Publikationen zeigten die Autoren der vorliegenden Studie durch die standardisierte Charité-Testbatterie einen subjektiven Benefit bei Patient:innen mit SSD und AHL nach CI-Versorgung in Hinblick auf Tinnitussuppression, Stressniveau und Lebensqualität (Abb. 1, [15–19]). Es existieren jedoch kaum Studien, in denen die beiden Patientengruppen vergleichend untersucht werden [20]. Darüber hinaus wird eine strikte Unterteilung zwischen AHL und SSD nicht konsequent in der Literatur vorgenommen [21]. Es bleibt also unklar, wie sich SSD- und AHL-Patient:innen präoperativ und postoperativ unterscheiden. Es wird vermutet, dass es sich bei SSD-Patient:innen häufig um eine akute Ertaubung oder einen Hörsturz handelt, während AHL-Patient:innen häufig einen schleichenden Hörverlust entwickeln. Darüber hinaus können die unterschiedlichen soziodemografischen Charakteristika (z. B. Alter) beider Gruppen einen Einfluss auf die psychischen Komorbiditäten der Patienten haben. Ziel der vorliegenden Studie ist die direkte Vergleichsanalyse beider Patientengruppen, um die gruppenspezifischen Bedürfnisse der Patient:innen besser charakterisieren und berücksichtigen zu können. In der vorliegenden Arbeit handelt sich um eine statistische Sekundäranalyse bereits veröffentlichter Daten über das CI-Outcome bei SSD- [17] und AHL-Patient:innen [15]. Hier werden die Parameter subjektive Höreinschätzung, Tinnitusbelastung, Lebensqualität und psychische Komorbiditäten (Stress, Ängstlichkeit, depressive Symptomatik) bei SSD- und AHL-Patient:innen direkt miteinander verglichen und statistisch ausgewertet, um einen möglichen Unterschied zwischen diesen Subgruppen zu identifizieren und bei der Rehabilitation zu adressieren.

## Methoden und Patienten

### Patientenkohorte

In die vorliegende Studie wurden 66 Patient:innen prospektiv eingeschlossen, die über einen 7‑Jahres-Zeitraum in der Klinik für Hals‑, Nasen‑, Ohrenheilkunde der Charité – Universitätsmedizin Berlin, Campus Virchow-Klinikum, operiert und mit einem CI versorgt worden sind.

Die Daten der SSD-Kohorte (*n* = 21) wurden bereits veröffentlicht [17]. Die vorliegende AHL-Kohorte (*n* = 45) entspricht nicht vollständig der veröffentlichten AHL-Kohorte von Ketterer et al. [15], wobei beide Kohorten partiell übereinstimmen.

Einschlusskriterien waren ein intakter Hörnerv, eine unauffällige Anatomie des Innenohrs und die postlinguale Ertaubung auf dem schlechter hörenden Ohr mit einem Sprachverstehen von ≤ 60 % mit Hörgerät im Freiburger Einsilbertest bei 65 dB SPL entsprechend der S2k-Leitlinie der Deutschen Gesellschaft für Hals-Nasen-Ohren-Heilkunde, Kopf- und Hals-Chirurgie, e. V. (DGHNO-KHC) [1]. Entsprechend dem Hörvermögen auf dem Gegenohr wurden die Patient:innen in 2 Gruppen unterteilt:

- „single-sided deafness“ (SSD, einseitige Ertaubung),
- „asymmetric hearing loss“ (AHL, asymmetrischer Hörverlust).

Bei der Gruppe mit SSD bestand eine sensorineurale Schwerhörigkeit von ≤ 30 dB im Reintonaudiogramm im besser hörenden Ohr und > 70 dB im schwächer hörenden Ohr [8]. Ein AHL lag vor bei sensorineuraler Schwerhörigkeit von > 30 und ≤ 70 dB im Reintonaudiogramm im besser hörenden Ohr und > 70 dB im schwächer hörenden Ohr [8].

Ausschlusskriterien waren ein Alter von unter 18 Jahren, eine Nachbeobachtungszeit von weniger als 6 Monaten, das Vorliegen einer Sprachbarriere, eine neuropsychiatrisch diagnostizierte kognitive Einschränkung. Es gab keine weiteren Einschränkungen bezüglich des Alters, der Ertaubungsdauer oder der Ausprägung der Tinnitusbelastung.

### Hörmessungen und Studienablauf

Vor der Operation erfolgte eine Tonschwellenaudiometrie, die Sprachaudiometrie mittels des Freiburger Einsilbertests (mit Hörgerät) und eine Befragung mittels validierter Fragebögen entsprechend der Charité-Testbatterie [15–17, 19]. Der Freiburger Einsilbertest wurde bei 65 dB ohne Störgeräusch durchgeführt. Die Anpassung des Sprachprozessors erfolgte 4 Wochen nach erfolgter CI-Operation. Zur postoperativen Verlaufskontrolle wurde 6 Monate nach der Erstanpassung der Freiburger Einsilbertest mit CI durchgeführt. Die Fragebögen wurden per Post zugesendet und von den Patient:innen selbstständig ausgefüllt. Von den in die Studie eingeschlossenen Patient:innen sind schriftliche Einverständniserklärungen zur Studienteilnahme vorhanden. Die Genehmigung der Ethikkommission der Charité – Universitätsmedizin Berlin zur Durchführung der Studie liegt vor (EA2/030/13). Die Studie wurde entsprechend der Deklaration von Helsinki durchgeführt. Die statistische Auswertung wurde mit der JMP 15 Software (Fa. SAS Institute Inc., Cary, NC, USA) durchgeführt. Die Gruppenunterschiede wurden als Box-Plots grafisch dargestellt. In den Grafiken entspricht die Box dem Interquartilsabstand, wobei der untere Rand der Box dem ersten Quartil (Q1) und der obere Rand dem dritten Quartil (Q3) entspricht. Die horizontale Linie innerhalb der Box entspricht dem Median, während die Whisker den Bereich der Daten außerhalb der Interquartilsabstände repräsentieren. Ausreißer werden durch einzelne Punkte dargestellt. Die statistische Gruppenanalyse wurde mittels des nichtparametrischen Wilcoxon-Rangsummentests durchgeführt, da die meisten Daten nicht normalverteilt waren (Shapiro-Wilk-Test). Um den Einfluss der konfundierenden Variablen zu untersuchen, wurde eine nichtparametrische Korrelationsanalyse durchgeführt, wobei die kausale Bedeutung dieser Faktoren nicht nachgewiesen werden kann. Die Korrelationsanalyse wurde mittels des Spearman-ρ-Koeffizienten durchgeführt. Das Signifikanzniveau wurde bei *p* < 0,05 festgelegt.

### Validierte Fragebögen

#### Oldenburger Inventar

Der Oldenburger-Inventar(OI)-Fragebogen erfasst das subjektive Hörvermögen und umfasst 12 Fragen [22]. Es gibt jeweils 5 Fragen zum Sprachverstehen in Ruhe und im Störgeräusch sowie 2 Items, die das Richtungshören abfragen. Die Punkte jeder Kategorie werden addiert. Je höher also der Wert, desto eher wird das Sprachverstehen als unbeeinträchtigt eingeschätzt.

#### Nijmegen Cochlear Implant Questionnaire

Der Nijmegen Cochlear Implant Questionnaire (NCIQ) ist ein Fragebogen, der speziell zur Evaluation der Lebensqualität von CI-Patient:innen entwickelt wurde [23]. Er besteht aus 60 Fragen, die in 3 Hauptdomänen und 6 Subdomänen „elementare Schallwahrnehmung“, „erweiterte Schallwahrnehmung“, „Sprachproduktion“, „Selbstwertgefühl“ „Aktivitätsverhalten“ und „soziale Interaktion“ eingeteilt werden, wobei die ersten 2 Subdomänen „elementare und erweiterte Schallwahrnehmung“ auch als Parameter der subjektiven Höreinschätzung betrachtet werden können. Je höher der erreichte Punktwert ist, desto näher schätzen sich die Patient:innen im Bereich der Normakusis ein.

#### Tinnitusfragebogen

Der Tinnitusfragebogen (Tinnitus Questionnaire, TF) [24] umfasst 52 Fragen, die den Bereichen „kognitive Beeinträchtigung“ (C = „cognitive distress“), „emotionale Beeinträchtigung“ (E = „emotional distress“), „Penetranz des Tinnitus“ (I = „intrusiveness“), „Hörprobleme“ (A = „auditory perceptual difficulties“), „Schlafstörungen“ (SI = „sleep disturbances“) und „somatische Beschwerden“ (So = „somatic disturbances“) angehören. Die Gruppen kognitive Beeinträchtigung und emotionale Beeinträchtigung werden auch zu „psychischer Beeinträchtigung“ zusammengefasst (C + E). Der maximale Gesamtscore liegt bei 84 Punkten. Es gibt 4 Schweregrade der Beeinträchtigung: leicht (0–30), mittelgradig (31–46), schwer (47–59) oder sehr schwer (60–84).

#### Perceived Stress Questionnaire

Die deutsche Version des Perceived Stress Questionnaire (PSQ) [25] enthält 20 Items und die Kategorien „Sorgen“, „Anspannung“, „Freude“ und „Anforderungen“. Alle Items sind als Aussagen, die sich auf die letzten 4 Wochen vor der Befragung beziehen, formuliert, denen mehr oder weniger zugestimmt werden soll. Da die Skala „Freude“ positiv formulierte Aussagen enthält, wird der Wert invers berechnet. Ein besonders hoher Wert steht also für ein hohes empfundenes Stresslevel. Werte zwischen 0,45 und 0,6 wurden als moderates Stressempfinden und Werte über 0,6 als hohes Stressempfinden festgelegt [25].

#### Generalized-Anxiety-Disorder-Fragebogen

Der Generalized-Anxiety-Disorder-Fragebogen (GAD-7) [26] ist ein Screening-Fragebogen für eine generalisierte Angststörung. Auf einer 7‑Punkte-Skala werden die Hauptsymptome einer generalisierten Angststörung in den letzten 2 Wochen vor der Befragung bewertet. Der maximale Gesamtscore liegt bei 21 Punkten. Es gibt 4 Schweregrade: minimal (0–4), leicht (5–9), mittel (10–14) und schwer (15–21).

#### Allgemeine Depressionsskala

Die Allgemeine Depressionsskala (ADS-L) von Hautzinger und Bailer umfasst 20 Fragen [27]. Ab einem Cut-off-Wert von 23 weist die ADS‑L auf eine depressive Symptomatik hin.

## Ergebnisse

### Patientenkohorte

Insgesamt wurden 66 CI-Patient:innen prospektiv in die vorliegende Untersuchung eingeschlossen (32 m. und 34 w.). Das durchschnittliche Alter betrug 59,4 ± 13,8 Jahre für die gesamte Patientenkohorte. Aufgrund der genannten Kriterien wurden 21 Patient:innen (8 m. und 13 w.) der SSD-Gruppe und 45 Patient:innen (24 m. und 21 w.) der AHL-Gruppe zugeteilt. Das durchschnittliche Alter in der AHL-Gruppe (62,6 ± 12,6 Jahre) war signifikant höher als in der SSD-Gruppe (52,6 ± 14,1 Jahre; Wilcoxon-Rangsummentest, *p* < 0,01; Abb. 1). Die durchschnittliche Ertaubungsdauer betrug 17,4 ± 21,3 Jahre in der AHL-Gruppe und 10,1 ± 15,5 Jahre in der SSD-Gruppe ohne statistisch signifikanten Gruppenunterschied (Wilcoxon-Test, *p* > 0,05; Abb. 1). Daten über soziale Parameter (z. B. Familiensituation, Berufstätigkeit) wurden in der vorliegenden Studie nicht erhoben. Hinsichtlich der Ätiologie zeigte die SSD-Gruppe die folgende Verteilung: Hörsturz (*n* = 8), Meningitis (*n* = 2), M. Menière (*n* = 2), Varizella-Zoster-Virus (*n* = 1), Trauma (*n* = 1) und unklar (*n* = 6).

### Hörvermessungen

Das präoperative Sprachverstehen für das implantatversorgte Ohr betrug 3,5 % ± 11,3 % im Freiburger Einsilbertest in der SSD-Gruppe und 3,3 % ± 8,8 % in der AHL-Gruppe (Tab. 1). Beide Gruppen zeigten 6 Monate nach CI eine signifikante Verbesserung (44,6 % ± 27,6 % für die SSD-Gruppe und 35,3 % ± 27,7 % für die AHL-Gruppe). Es gab keine signifikanten Unterschiede zwischen beiden Gruppen hinsichtlich des Sprachverstehens prä- oder postoperativ (Tab. 1). In der vorliegenden Studie wurde eine statistische Vergleichsanalyse zwischen SSD- und AHL-Patient:innen durchgeführt. Der Einfluss der CI-Versorgung auf die einzelnen Gruppen wurde bereits von der Arbeitsgruppe untersucht und veröffentlicht [15–17, 19], eine signifikante Verbesserung der subjektiven Höreinschätzung, krankheitsspezifischen Lebensqualität, Tinnitusbelastung und Stressbelastung durch CI wurde gezeigt [15–17, 19].

**Table 1:** 

	SSD	AHL	*p*-Wert
Freiburger Einsilbertest präoperativ	MW (± SD): 3,5 % (± 11,3)	MW (± SD): 3,3 % (± 8,8)	0,62
	Median: 0 %	Median: 0 %	
	Min.: 0 %	Min.: 0 %	
	Max.: 50 %	Max.: 40 %	
Freiburger Einsilbertest postoperativ	MW (± SD): 44,6 % (± 27,6)	MW (± SD): 35,3 % (± 27,7)	0,19
	Median: 52,5 %	Median: 35 %	
	Min.: 0 %	Min.: 0 %	
	Max.: 75 %	Max.: 90 %	
OI „Hören in Ruhe“ präoperativ	MW (± SD): 3,9 % (± 0,7)	MW (± SD): 3,4 % (± 0,9)	0,07
	Median: 4 %	Median: 3,5 %	
	Min.: 1,8 %	Min.: 1,8 %	
	Max.: 5 %	Max.: 5 %	
OI „Hören im Störgeräusch“ präoperativ	MW (± SD): 2,7 % (± 0,6)	MW (± SD): 2,6 % (± 0,9)	0,37
	Median: 2,6 %	Median: 2,4 %	
	Min.: 1,4 %	Min.: 1 %	
	Max.: 3,8 %	Max.: 5 %	
OI „Richtungshören“ präoperativ	MW (± SD): 1,9 % (± 1,0)	MW (± SD): 2,4 % (± 1,0)	0,10
	Median: 2 %	Median: 2 %	
	Min.: 0 %	Min.: 1 %	
	Max.: 3,5 %	Max.: 5 %	
OI gesamt präoperativ	MW (± SD): 3,1 % (± 0,5)	MW (± SD): 2,9 % (± 0,8)	0,21
	Median: 3,17 %	Median: 2,96 %	
	Min.: 2,08 %	Min.: 1,67 %	
	Max.: 3,92 %	Max.: 5 %	
OI „Hören in Ruhe“ Postoperativ	MW (± SD): 4,2 % (± 0,6)	MW (± SD): 3,9 % (± 0,6)	*0,04*
	Median: 4,5 %	Median: 4 %	
	Min.: 2,6 %	Min.: 2,4 %	
	Max.: 5 %	Max.: 5 %	
OI „Hören im Störgeräusch“ postoperativ	MW (± SD): 3,1 % (± 0,7)	MW (± SD): 3,1 % (± 0,7)	0,82
	3,3 (1,6–4,2)	3 (1,8–5)	
	Median: 3,3 %	Median: 3 %	
	Min.: 1,6 %	Min.: 1,8 %	
	Max.: 4,2 %	Max.: 5 %	
OI „Richtungshören“ postoperativ	MW (± SD): 2,9 % (± 1,1)	MW (± SD): 3,2 % (± 0,9)	0,42
	Median: 3 %	Median: 3,5 %	
	Min.: 1 %	Min.: 1 %	
	Max.: 4,8 %	Max.: 5 %	
OI gesamt postoperativ	MW (± SD): 3,4 % (± 0,7)	MW (± SD): 3,4 % (± 0,6)	0,63
	Median: 3,66 %	Median: 3,34 %	
	Min.: 2,08 %	Min.: 2,08 %	
	Max.: 4,4 %	Max.: 5 %	

Die Daten sind als Mittelwert ± Standardabweichung (MW ± SD) sowie als Median, Minimum und Maximum dargestellt

Die Gruppenunterschiede und *p*-Werte wurden durch den Wilcoxon-Rangsummentest evaluiert

*SSD *einseitige Taubheit („single-sided deafness“), *AHL* asymmetrischer Hörverlust („asymmetric hearing loss“)

### Subjektive Höreinschätzung

**Figure 2:**
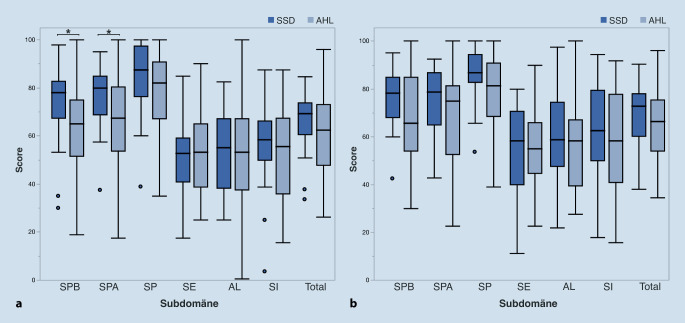

Hinsichtlich der subjektiven Einschätzung des Hörvermögens zeigte sich präoperativ kein statistischer signifikanter Unterschied zwischen den Gruppen im Oldenburger Inventar (OI) bei „Hören in Ruhe“ zugunsten der SSD-Gruppe (Wilcoxon-Test, *p* = 0,07). Postoperativ zeigte dieser Parameter 6 Monate nach CI-Versorgung einen signifikanten Unterschied zwischen den Gruppen zugunsten der SSD-Gruppe (*p* < 0,05). Dieses Ergebnis könnte darauf hinweisen, dass die postoperative Verbesserung der subjektiven Höreinschätzung in Ruhe bei AHL-Patient:innen stärker ausgeprägt war. Die anderen Parameter des OI zeigten keine signifikanten Unterschiede zwischen beiden Gruppen (*p* > 0,05; Tab. 1). Die ersten 2 Subdomänen des NCIQ-Fragebogens „elementare Schallwahrnehmung“ und „erweiterte Schallwahrnehmung“ reflektieren auch die subjektive Höreinschätzung der Patienten. Die NCIQ-Subdomäne „elementare Schallwahrnehmung“ war präoperativ in der SSD-Gruppe (73,2 ± 16,7) signifikant höher als in der bimodal versorgten AHL-Gruppe (62,1 ± 19,9; Wilcoxon-Rangsummentest, *p* < 0,05; Abb. 2). Ebenso war die NCIQ-Subdomäne „erweiterte Schallwahrnehmung“ präoperativ in der SSD-Gruppe (77,0 ± 13,6) signifikant höher als in der AHL-Gruppe (66,1 ± 19,3; *p* < 0,05). Diese Ergebnisse sind mit einer besseren subjektiven Höreinschätzung bei SSD-Patienten vereinbar.

### Krankheitsspezifische Lebensqualität

Die krankheitsspezifische Lebensqualität (NCIQ) wurde durch den NCIQ-Fragebogen bewertet. Die Subdomänen „Sprachproduktion“, „Selbstwertgefühl“, „Aktivitätsverhalten“ und „soziale Kontakte“ zeigten präoperativ keine signifikanten Unterschiede zwischen den SSD- und AHL-Gruppen (*p* > 0,05; Abb. 2). Postoperativ gab es in keiner der NCIQ-Subdomänen Unterschiede zwischen der SSD- und der AHL-Gruppe (*p* > 0,05; Abb. 2). Die Korrelationsanalyse zeigte eine signifikante negative Korrelation zwischen Alter und postoperativem NCIQ-Score „Sprachproduktion“, „Aktivitätsverhalten“ und Gesamtscore (Spearman-ρ-Koeffizient, *p* < 0,05). Es zeigte sich auch eine signifikante Korrelation zwischen Ertaubungsdauer und postoperativem NCIQ-Score „Sprachproduktion“ (*p* < 0,05) und eine Tendenz zur Signifikanz zwischen Ertaubungsdauer und postoperativem Score „soziale Kontakte“ (*p* = 0,055), zwischen Ertaubungsdauer und postoperativem Score „Selbstwertgefühl“ (*p* = 0,06) sowie zwischen Ertaubungsdauer und Gesamtscore (*p* = 0,06).

### Tinnitusbelastung (Tinnitusfragebogen)

**Figure 3:**
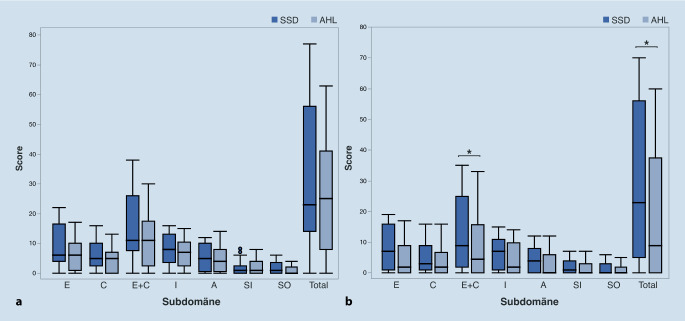

Präoperativ betrug der durchschnittliche TF-Gesamtscore 32,4 (±25,2) in der SSD-Gruppe (entsprechend einer mittelgradigen Tinnitusbelastung) und 25,8 (±19,5) in der AHL-Gruppe (entsprechend einer leichten Tinnitusbelastung) [24]. Die präoperativen Gesamtscore-Werte und Subdomänen unterschieden sich zwischen beiden Gruppen nicht signifikant (Wilcoxon-Rangsummentest, *p* > 0,05; Abb. 3). Bei der SSD-Gruppe bestanden 6 Monate nach CI-Versorgung signifikant höhere Werte im Bereich der psychischen Beeinträchtigung (E + C) und ein signifikant höherer Gesamtscore (*p* < 0,05). Dieses Ergebnis könnte darauf hinweisen, dass AHL-Patient:innen eine stärkere Tinnitussuppression postoperativ erfahren haben.

### Stressbelastung

**Figure 4:**
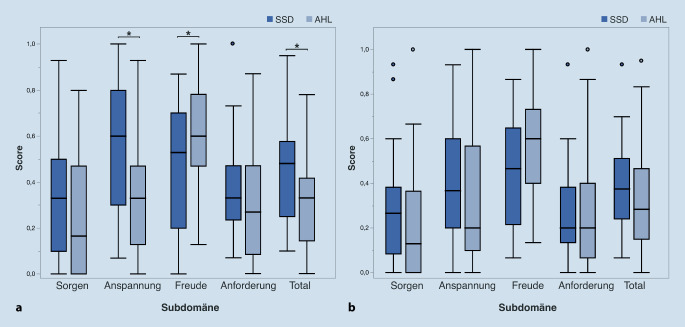

Der Stressbelastungs(PSQ)-Gesamtwert der SSD-Patientenkohorte (0,45) war präoperativ erhöht im Vergleich zur allgemeinen Bevölkerung (0,33) [25] und wird als moderate Stressbelastung eingeteilt. Dagegen war der präoperative PSQ-Gesamtwert der AHL-Kohorte (0,31) in dem Bereich für gesunde Erwachsene. Der präoperative PSQ-Gesamtwert und der Wert der Subdomäne „Anspannung“ waren in der SSD-Gruppe signifikant höher als in der AHL-Gruppe (Wilcoxon-Rangsummentest, *p* < 0,05; Abb. 4). Dahingegen war der präoperative Wert der Subdomäne „Freude“ bei der SSD-Gruppe signifikant geringer als bei der AHL-Gruppe (*p* < 0,05). Diese Ergebnisse weisen darauf hin, dass SSD-Patienten präoperativ mehr Anspannung und weniger Freude empfinden als AHL-Patienten. Postoperativ näherten sich die PSQ-Werte der SSD-Gruppe denen der AHL-Gruppe an, sodass postoperativ kein signifikanter Unterschied zwischen den Gruppen nachweisbar war (*p* > 0,05; Abb. 4). Die Korrelationsanalyse zeigte eine signifikante negative Korrelation zwischen Alter und präoperativem PSQ-Gesamtscore (Spearman-ρ = −0,29; *p* < 0,05) und eine Tendenz zur Signifikanz zwischen Alter und postoperativem PSQ-Gesamtscore (Spearman-ρ = −0,24; *p* = 0,052).

### Ängstlichkeit

**Figure 5:**
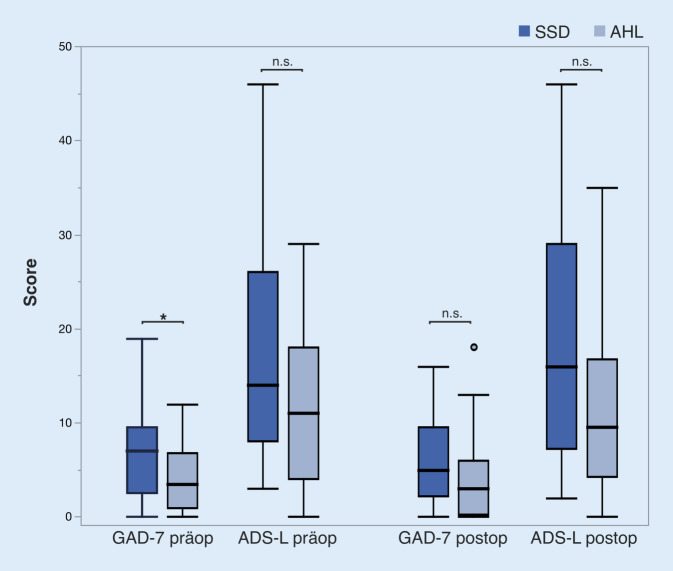

Präoperativ war die Ängstlichkeit oder Angstsymptomatik, gemessen durch die GAD-7-Werte, bei der SSD-Gruppe signifikant höher als die der AHL-Gruppe (*p* < 0,05). Der präoperative GAD-7-Wert bei der SSD-Gruppe (7,0) entspricht einer „leichten“ Angstsymptomatik, während der präoperative Wert der AHL-Gruppe (4,0) einer „minimalen“ Angstsymptomatik entspricht. Postoperativ haben sich die GAD-7-Werte beider Gruppen angeglichen, sodass der Unterschied nicht mehr signifikant war (*p* > 0,05; Abb. 5a).

### Depressive Symptomatik

Bezüglich der depressiven Symptomatik, gemessen mittels ADS‑L, war der durchschnittliche präoperative ADS-L-Score bei beiden Gruppen unter dem Cut-off-Wert von 23. Die präoperativen ADS-L-Werte der SSD-Patient:innen waren nahezu signifikant höher als die der AHL-Patient:innen (*p* = 0,052; Abb. 5b). Diese Tendenz blieb postoperativ vorhanden (*p* = 0,056), jedoch wurde eine statistische Signifikanz auch hier nicht erreicht.

## Diskussion

Die CI-Versorgung bei SSD- und AHL-Patient:innen hat sich in den letzten Jahren etabliert und ist ebenfalls in die S2k-Leitlinie der DGHNO-KHC aufgenommen worden [1]. Bei AHL wird das kontralaterale Ohr zusätzlich mit einem Hörgerät versorgt, sodass eine bimodale Hörrehabilitation erreicht wird. Bisher bestand die mögliche Therapie bei SSD und AHL in der Behandlung mit CROS- bzw. BiCROS-Hörgeräten („contralateral routing of signals“ bzw. „bilateral CROS“), die Wiederherstellung des binauralen Hörens ist jedoch dadurch nicht möglich [28]. Dagegen ermöglicht die CI-Versorgung ein binaurales Hören mit verbesserter Schalllokalisierung, verringerter Tinnitusbelastung und einer Steigerung der Lebensqualität [29]. In der vorliegenden Studie konnten bereits präoperativ signifikante Unterschiede zwischen SSD- und AHL-Patient:innen im Hinblick auf die untersuchten Parameter gezeigt werden. Diese Signifikanz war nach einer Beobachtungszeit von 6 Monaten im Anschluss an die CI-Versorgung stark verringert oder teils sogar nicht mehr gegeben. Die dargestellten Ergebnisse unterstützen den Ansatz, eine Unterscheidung zwischen den Patientengruppen mit SSD und AHL vorzunehmen und diese getrennt zu betrachten.

Eine Limitation der vorliegenden Studie ist die fehlende Charakterisierung der bimodalen Stimulation bei AHL-Patient:innen (Art und Anpassungsdetails der Hörgeräteversorgung). Die binauralen Hörfähigkeiten wurden in der vorliegenden Studie nicht geprüft. Außerdem ist die relativ kurz gewählte Nachbeobachtungszeit von 6 Monaten postoperativ zu erwähnen. Allerdings liegt der Fokus der vorliegenden Vergleichsstudie insbesondere auf der Betrachtung der Gruppenunterschiede zwischen SSD- und AHL-Patient:innen. Eine weitere Limitation ist, dass die Daten der Sozialanamnese (z. B. Berufstätigkeit) in der vorliegenden Studie nicht erhoben wurden. Es ist also möglich, dass es weitere Faktoren geben könnte, die zu den beobachteten Gruppenunterschieden beigetragen haben könnten. Daher kann die Möglichkeit von weiteren Konfundierungen nicht ausgeschlossen werden.

### Hörvermögen

Die CI-Versorgung von SSD- und AHL-Patient:innen führt zu einer Hörverbesserung, sowohl in Ruhe als auch im Störgeräusch, wie auch bereits in mehreren Studien gezeigt wurde [15–17, 19]. In der vorliegenden Studie konnte ein durchschnittliches Sprachverstehen von 44,6 % bei der SSD-Gruppe und 35,3 % bei der AHL-Gruppe 6 Monate postoperativ erreicht werden. Bei der letzten Kontrolluntersuchung (12 Monate postoperativ) wurde ein durchschnittliches Sprachverstehen von 48,5 % bei der SSD-Gruppe und 51,7 % bei der AHL-Gruppe erreicht. Diese Hörergebnisse sind zwar unter der leitliniengerechten Indikationsgrenze (60 % Einsilberverstehen), allerdings betrug der Median des Sprachverstehens (12 Monate postoperativ) 60 % bei der SSD-Gruppe und 53,75 % bei der AHL-Gruppe, sodass bei den meisten Patienten der vorliegenden Studie von einer erfolgreichen Hörrehabilitation ausgegangen wird. Eine weitere Verbesserung der Hörergebnisse bis 36 Monate postoperativ kann auch häufig erwartet werden. Die Hörrehabilitation mit CI wird als aktiver Lernprozess betrachtet, der sogar Jahre dauern kann [30]. Bei zukünftigen Studien sollen die Hörergebnisse und psychosozialen Parameter nach längeren Nachbeobachtungszeiten untersucht werden.

### Subjektive Höreinschätzung

Die subjektive Höreinschätzung wurde in mehreren vorangegangenen Arbeiten durch den Fragebogen Speech, Spatial and Qualities of Hearing Scale (SSQ) bewertet [20, 31]. In der vorliegenden Arbeit wurden diese Aspekte bei SSD- und AHL-Patienten mittels der gleichnamigen Unterkategorien des OI evaluiert. Darüber hinaus wurden sowohl die NCIQ-Subdomänen „elementare Schallwahrnehmung“ und „erweiterte Schallwahrnehmung“ auch als Parameter der subjektiven Höreinschätzung verwendet. In der vorliegenden Studie zeigten die SSD-Gruppe präoperativ signifikant höhere NCIQ-Subdomänen „elementare Schallwahrnehmung“ und „erweiterte Schallwahrnehmung“ als die AHL-Gruppe. Ansonsten können die vergleichbaren präoperativen Ergebnisse des Freiburger Einsilbertests und des OI bei SSD- und AHL-Patienten die signifikanten Unterschiede in den Daten zum Stress- und Ängstlichkeitsniveau zwischen den beiden Gruppen allerdings nicht suffizient begründen. Ein Erklärungsansatz könnte die kürzere durchschnittliche Ertaubungsdauer in der SSD-Gruppe sein (10,1 vs. 17,4 Jahre), welche sich in einer verringerten Zeit für die Adaptation und Kompensation der einseitigen Ertaubung äußern könnte, wobei der Unterschied in der Ertaubungsdauer zwischen beiden Gruppen in der vorliegenden Studie aufgrund von Ausreißern in der SSD-Gruppe (mit sehr langer Ertaubungsdauer) nicht signifikant war. Die AHL-Patientenkohorte war auch signifikant älter als die SSD-Kohorte (62 Jahre vs. 52 Jahre). Dieser Altersunterschied könnte von Bedeutung sein, da die Stressbelastung in der Bevölkerung (gemessen durch die PSQ-Werte) ihren Höhepunkt in der Altersgruppe „35–54 Jahre“ erreicht und dann mit zunehmendem Alter stetig abnimmt [32]. Ein weiterer Erklärungsansatz ist die unterschiedliche Ursache der Ertaubung. Bei SSD handelt es sich häufig um eine akute Ertaubung (z. B. im Rahmen eines Hörsturzes), während AHL-Patienten häufig einen schleichenden Hörverlust entwickeln. Da die Daten über die Ursache der Ertaubung in der AHL-Gruppe nicht vollständig vorliegen, konnte diese Hypothese nicht statistisch geprüft werden. In der vorliegenden Studie gaben 8 der 21 SSD-Patienten (38,1 %) an, einen Hörsturz erlitten zu haben. Die akute Ertaubung bei SSD-Patienten könnte die Angst (und den Stress) vor einem Hörverlust auf der zweiten Seite auslösen. Aufgrund der geringen Patientenanzahl konnte keine weitere Analyse der Rolle der Ertaubungsursache bei SSD-Patienten durchgeführt werden.

### Krankheitsspezifische Lebensqualität

Dillon et al., [13] haben die Verbesserung der Lebensqualität bei SSD-Patient:innen durch CI im Rahmen einer systematischen Übersicht von 21 Studien gezeigt. In vorhergehenden Studien zeigten die Autoren der vorliegenden Studie die Verbesserung der gesundheitsbezogenen Lebensqualität durch CI bei SSD- [17, 19] und AHL-Patient:innen [15, 16], jedoch wurden beide Gruppen dabei nicht vergleichend untersucht. In der vorliegenden Studie unterschieden sich die NCIQ-Subdomänen „Sprachproduktion“, „Selbstwertgefühl“, „Aktivitätsverhalten“ und „soziale Interaktion“ nicht signifikant. Da das Alter und die Ertaubungsdauer teilweise mit der krankheitsspezifischen Lebensqualität korrelieren, könnten diese Faktoren eine kausale Bedeutung haben. Es kann also nicht ausgeschlossen werden, dass die beobachteten Gruppenunterschiede auch auf unterschiedliche Altersverteilung und Ertaubungsdauer zurückzuführen sind. Zur besseren Vergleichbarkeit sollen beide Patientengruppen bei zukünftigen Studien durch einen Matching-Prozess untersucht werden, um den konfundierenden Einfluss der soziodemografischen Störfaktoren besser zu kontrollieren.

### Tinnitusbelastung

In der vorliegenden Arbeit wurde die Tinnitusbelastung durch den Tinnitusfragebogen (TF) nach Goebel und Hiller [24] evaluiert. Präoperativ zeigten sich keine signifikanten Unterschiede zwischen SSD- und AHL-Patient:innen hinsichtlich der TF-Subdomänen oder des Gesamtscores. Postoperativ zeigte die SSD-Gruppe signifikant höhere Werte im Bereich der psychischen Beeinträchtigung (E + C) und einen signifikant höheren Gesamtscore. Dieses Ergebnis könnte darauf hinweisen, dass AHL-Patient:innen eine stärkere Tinnitussuppression durch CI erfahren. Sydlowski et al. [20] zeigten die signifikant höhere präoperative Tinnitusbelastung (gemessen durch das Tinnitus-Handicap-Inventar) bei SSD-Patient:innen im Vergleich zu AHL-Patient:innen, wobei die Ertaubungsdauer der SSD-Gruppe in der Studie von Sydlowski (3 Jahre) deutlich kürzer als die Ertaubungsdauer der SSD-Patienten der vorliegenden Studie war (10,1 Jahre). Insgesamt bestätigen die Ergebnisse die Evidenz in der Literatur für den positiven Einfluss der CI-Versorgung auf die Tinnitussuppression bei SSD-Patient:innen. Eine aktuelle systematische Übersicht von 17 Studien bei SSD-Patient:innen ergab eine hohe Wahrscheinlichkeit (75–100 %) der signifikanten Reduktion der Tinnitusbelastung durch CI [13].

### Stressbelastung, Ängstlichkeit und depressive Symptomatik

Die Beobachtung, dass SSD-Patient:innen eine subjektiv stärkere Einschränkung in Hinblick auf Stressbelastung und Ängstlichkeit haben, ist von großer Bedeutung. SSD-Patient:innen werden in ihrem sozialen Umfeld in der Regel nicht als so stark (hör)beeinträchtigt wahrgenommen wie AHL-Patient:innen, die mit einem Hörgerät versorgt sind. Sydlowski et al. zeigten, dass AHL- und SSD-Patient:innen ähnliche subjektive Einschränkung in Hinblick auf die auditive Fähigkeit haben, jedoch wurden die psychischen Komorbiditäten und Lebensqualität dabei nicht untersucht [20]. In der vorliegenden Studie stellt die Vergleichsanalyse der psychischen Komorbiditäten zwischen beiden Gruppen eine wichtige Ergänzung dar, da die stärkeren psychosozialen Einschränkungen der SSD-Patient:innen in den erhobenen Daten gezeigt werden konnten.

Generell ist die Wiederherstellung des binauralen Hörens bei jedem einzelnen Patienten erstrebenswert, unabhängig von dem individuellen Hörstatus des kontralateralen Ohrs. Schlussendlich profitierten beide Gruppen signifikant von einer CI-Versorgung, allerdings ermöglicht die komplexe Outcome-Messung mittels validierter Fragebögen, die gruppenspezifischen Aspekte der Patient:innen zu charakterisieren und berücksichtigen. Vor allem bei SSD könnte die psychologische Betreuung der Patient:innen während der ersten Monate der CI-Rehabilitation eine Verbesserung der subjektiven Ergebnisse im Rahmen der Hörrehabilitation ermöglichen. Wenn eine bereits präoperativ vorhandene psychische Störung festgestellt wird, sollte vor CI eine entsprechende Psychotherapie eingeleitet werden. Ansonsten kann eine postoperative Counseling-Therapie den Patienten helfen, sich auf die Veränderungen vorzubereiten, die mit der CI-Versorgung einhergehen. Die Durchführung weiterer Studien auf diesem Gebiet ist anzustreben, sodass diese CI-Kandidaten bestmöglich präoperativ identifiziert und beraten sowie postoperativ engmaschig betreut werden können, um eine optimale individuelle Hörrehabilitation zu gewährleisten.

## Fazit für die Praxis

- Präoperativ unterscheiden sich Patient:innen mit einseitiger Taubheit („single-sided deafness“, SSD) und Patient:innen mit asymmetrischem Hörverlust („asymmetric hearing loss“, AHL) signifikant im Hinblick auf die subjektive Höreinschätzung und psychosoziale Parameter.
- AHL- und SSD-Patient:innen profitieren über die Verbesserung ihrer auditiven Fähigkeiten hinaus von der Cochleaimplantat(CI)-Versorgung.
- Bei SSD-Patient:innen können psychische Belastungsfaktoren einen stärkeren Einfluss auf die Lebensqualität haben als bei AHL-Patient:innen.
- Die Besonderheiten sollten in der Anamnese, der Therapieberatung und im Rehabilitationsprozess Beachtung finden.
- Ziel zukünftiger Studien sollte es sein, genaueren Aufschluss darüber zu geben, ob bei größeren Kollektiven diese Unterschiede weiterhin nachweisbar sind.
